# Origin and Evolution of Ammonium Transporters (AMTs) in Plants and Systematic Expression Studies in *Brassica napus*

**DOI:** 10.3390/plants15152334

**Published:** 2026-07-29

**Authors:** Mingming Chen, Li Zhang, Yixuan Zhang, Xingzhi Qian, Qingyang Yang, Zhuo Chen, Huiyan Zhao, Nengwen Yin, Ti Zhang, Cunmin Qu, Jing Wen, Hai Du, Daixiang Xu

**Affiliations:** 1 Integrative Science Center of Germplasm Creation in Western China (CHONGQING) Science City, College of Agronomy and Biotechnology, Southwest University, Chongqing 400715, Chinazhangti@swu.edu.cn (T.Z.); drqucunmin@swu.edu.cn (C.Q.);; 2 Academy of Agricultural Sciences, Southwest University, Chongqing 400715, China

**Keywords:** ammonium transporter (AMT), evolution, expression profile, yield, *Brassica napus*

## Abstract

*Brassica napus* L. is one of the most important oil crops in the world, and improving its nitrogen efficiency is a key strategy for enhancing low-nitrogen tolerance and increasing yield. Ammonium transporters (AMTs) play critical roles in NH_4_^+^ transport in plants and significantly influence nitrogen use efficiency and yield. To identify promising candidate *AMT* genes for rapeseed improvement, this study systematically analyzed the evolution and expression of *AMT* genes across the Archaeplastida plants, especially in *Brassica* species of the U’s triangle. A total of 368 *AMT* genes were identified from 35 Archaeplastida species, including 180 *AMT1* and 188 *AMT2* homologs. Gene duplication analysis revealed that small-scale duplication (SSD) was the primary mechanism driving gene expansion from aquatic algae to angiosperms in both *AMT1* and *AMT2* families, which were under the influence of purifying selection. In the U’s triangle, allopolyploidization, particularly whole-genome duplication (WGD), was the primary mechanism for *AMT* expansion, accompanied by gene loss. Expression profiling indicated that most *BnAMTs* respond to hormonal and low-nitrogen signals and exhibit tissue-specific expression patterns. Notably, four candidate genes (*BnAMT1;5*, *BnAMT1;12*, *BnAMT2;2*, *BnAMT2;4*) exhibited high expression levels and responsiveness to low-nitrogen stress. Haplotype analysis further linked specific polymorphisms in *BnAMT1;5* and *BnAMT2;2* to variations in plant height and yield. Collectively, these findings elucidate the evolutionary processes and regulatory mechanisms of *AMTs*, laying a foundation for enhancing nitrogen utilization efficiency in *B. napus* and other crops.

## 1. Introduction

Nitrogen (N), mainly absorbed from the soil, is an essential nutrient for plant growth and development. The sources of N include nitrate, ammonium (NH_4_^+^), amino acids, and other nitrogen-containing compounds [1,2]. In flooded soils where both ammonium and nitrate are present, ammonium is preferentially taken up by plants, as its assimilation is energetically less costly than that of nitrate [3,4]. This preference is especially pronounced under nitrogen deficiency [5,6]. NH_4_^+^ serves as a key nitrogen source for plants and participates in various physiological processes, such as pH homeostasis, gene regulation, and root development [7]. The assimilation of NH_4_^+^ into glutamine is mediated by glutamine synthetase (GS), which is localized in the cytoplasm and plastids [8]. Nevertheless, excessive ammonium is toxic to plants and inhibits their growth [1,9]. However, maintaining cellular NH_4_^+^ homeostasis is essential for normal plant growth and development.

Ammonium transporters (AMTs) are critical for ammonium uptake and transport in plants [10] and are mainly divided into two subfamilies: *AMT1* and *AMT2* [11,12]. *AMT1* members function as high-affinity ammonium transporters [10], whereas *AMT2* members are involved in long-distance ammonium transport [13]. To date, *AMT* genes have been identified in numerous plant species, including *Arabidopsis thaliana* [14], *Lycopersicon esculentum* [15], *Lotus japonicus* [16], *Brassica napus* [17], *Oryza sativa* [18,19], *Zea mays* [20], and *Sorghum bicolor* [21]. Different *AMT* members exhibit functional divergence across species, influencing nitrogen use efficiency, disease resistance, and yield in various crops [22,23,24,25]. However, the evolutionary relationships among *AMT* family members remain unknown, highlighting the need for a comprehensive analysis and classification across plant lineages.

*Brassica* species have diverse uses, including as animal fodder, vegetable oil, and biofuel resources [26,27]. The “U’s triangle” theory describes the hybridization among three ancestral diploid species: *Brassica rapa* (B. rapa, Bra, AA, 2n=20), *Brassica nigra* (*B. nigra*, *Bni*, *BB*, 2n = 16), and *Brassica oleracea* (*B. oleracea*, *Bol*, *CC*, 2n = 18), which gave rise to three allotetraploid species: *Brassica juncea* (*B. juncea*, *Bju*, *AABB*, 2n = 36), *Brassica napus* (*B. napus*, *Bna*, *AACC*, 2n = 38), and *Brassica carinata* (*B. carinata*, *Bca*, *BBCC*, 2n = 34) [28,29]. The availability of whole-genome sequencing and pan-genome data for these six *Brassica* species provides a practical evolutionary model for gene family studies [30,31]. *B. napus* is an important oil crop with high nitrogen demand and exhibits marked sensitivity to NH_4_^+^ levels [32,33]. Although *AMT* genes have been identified in *B. napus*, their function have not been thoroughly characterized, and their potential for improving nitrogen use efficiency and ammonium tolerance remains largely unexplored. Furthermore, the origin and evolutionary dynamics of *AMT* genes within the U’s triangle species require systematic investigation across diverse varieties.

In this study, we systematically identified 368 *AMT* genes across 35 representative Archaeplastida species, ranging from aquatic green algae to angiosperms. We characterized the distribution and evolutionary dynamics of *AMT* family members across the plant kingdom, with a specific focus on the U’s triangle species. To identify candidate *BnAMT* genes with potential for yield improvement, we analyzed their regulatory mechanisms and constructed expression profiles under spatiotemporal, hormonal, and low-nitrogen (L-N) conditions. Furthermore, through haplotype analysis, we identified distinct single nucleotide polymorphism (SNP) loci in *BnAMT1;5* and *BnAMT2;2* that significantly correlate with plant height and yield in a rapeseed population. Collectively, this study provides a foundation for improving nitrogen use efficiency and breeding ammonium-tolerant rapeseed cultivars.

## 2. Results

### 2.1. Identification and Distribution of AMT Genes in the Plant Kingdom

**Figure 1:**
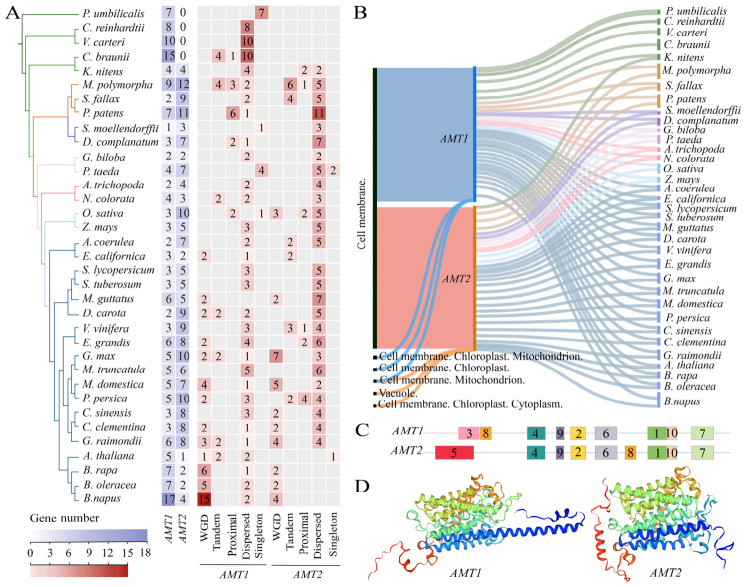
(**A**) Phylogenetic relationship and *AMT* gene number of 35 species. The phylogenetic tree on the left was built of 35 species spanning from green algae to angiosperms, with branches colored by major clades. Phylogenetic relationships (branch lengths are arbitrary) among these species have been described previously (https://phytozome-next.jgi.doe.gov/, accessed on 1 March 2026). The purple heatmap indicates *AMT1* and *AMT2* gene number in each species. The red heatmap displays gene duplication types. WGD: whole-genome duplication; tandem: tandem duplication; proximal: proximal duplication; dispersed: dispersed duplication; singleton: single gene. (**B**) Subcellular localization prediction of *AMT* genes in 35 species. (**C**) Schematic of conserved domains of the *AMT* gene family. (**D**) 3D protein structure prediction map of the *AMT* gene family.

To investigate the origin, evolution, and expansion history of ammonium transporter (*AMT*) genes, we identified 368 *AMT* genes across 35 representative Archaeplastida species based on the Phytozome v13 database, ranging from aquatic algae to angiosperms (Figure 1A, Table S1). Based on sequence homology with *AMT* genes of *A. thaliana*, these genes were classified into two subfamilies: *AMT1* subfamily (180 members) and *AMT2* subfamily (188 members). Gene nomenclature was assigned according to chromosomal physical positions within each species.

The number of *AMT1* genes varied considerably across evolutionary lineages. Algal species, with the exception of *Klebsormidium nitens*, harbored relatively more *AMT1* genes (7–15 per genome), whereas most land plant species (25 of 35 examined) contained only 1–6 copies (Figure 1A). Notably, *Marchantia polymorpha*, *Physcomitrella patens*, and three *Brassica* species (*Brassica oleracea* ‘Capitata’, *Brassica rapa* ‘FPsc’, *Brassica napus* ‘ZS11’) each contained seven or more *AMT1* genes, with the highest number (17) observed in *B. napus*. In contrast to *AMT1*, *AMT2* genes were absent in most algal species, with the exception of *Klebsormidium*, which possessed four *AMT* copies. Bryophytes contained the highest *AMT2* numbers (9–12 genes), whereas most land plants (22 species) had fewer copies (1–8 genes). In the majority of angiosperms, *AMT2* copy numbers exceeded those of *AMT1*. Notably, a marked reduction in *AMT2* copy number was observed in Brassicaceae.

Subcellular localization analysis revealed that most AMTs were predicted to localize to the plasma membrane, whereas a minority were localized to the mitochondria, chloroplasts, and cytoplasm (Figure 1B). Only AtrAMT2;3 was predicted to be in vacuoles. This diverse localization indicated that AMTs mediate ammonium transport mainly in the cell membrane, but also function at different cellular sites in different species. Ten conserved amino acid motifs were identified among the 180 AMT1 and 188 AMT2 proteins (Figure 1C). Eight of these motifs (Motif 8, 4, 9, 2, 6, 1, 10 and 7) were shared between the two subfamilies, and were predominantly located in the middle and C-terminal regions. Family-specific motifs were observed in the N-terminal regions of AMT1 and AMT2 (Figure 1D).

### 2.2. Evolution of AMT Genes in the Plant Kingdom

**Figure 2:**
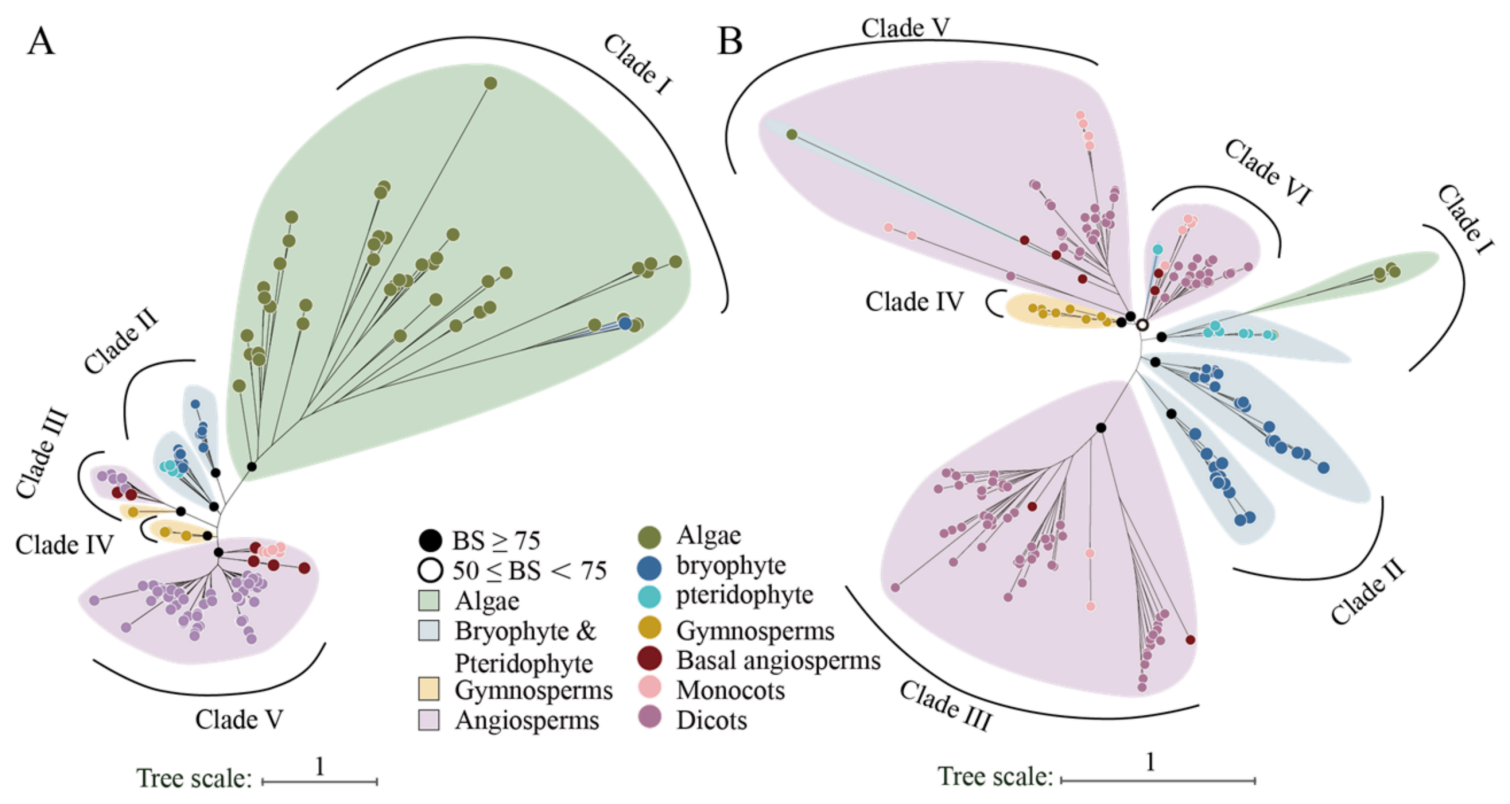
The phylogenetic tree and classification of the *AMT* gene family. (**A**) The maximum likelihood (ML) tree which includes 180 AMT1 proteins. (**B**) The ML tree which includes 188 AMT2 proteins. Bootstrap support (BS) ≥ 75% is shown as filled circles (●), 50–75% are shown as open circles (○), while <50% are not shown. The background shading symbolizes major plant groups. The colored dots represent the species to which the proteins in each clade belong.

We constructed two maximum likelihood (ML) phylogenetic trees based on multiple sequence alignments of the 180 AMT1 and 188 AMT2 protein sequences, respectively (Figure 2, Table S2). Based on the tree topologies and bootstrap support values, the 180 AMT1 and 188 AMT2 proteins were classified into five and six clades, respectively. In the *AMT1* family tree, Clade I contained all alga proteins and one bryophyte protein, MpAMT1;9. Clade II contained the remaining bryophyte and pteridophyte proteins. Clade III contained 13 angiosperm proteins and one gymnosperm protein (GibiAMT1;2). Clade IV contained gymnosperm proteins, whereas Clade V contained angiosperm proteins. The number of AMT1 proteins gradually decreased from Clade I to IV, but increased substantially in Clade V. In the *AMT2* family tree, all bryophyte proteins were located in Clade II. Within this clade, liverwort proteins occupied one branch, whereas proteins from *Physcomitrella patens* and *Sphagnum fallax* jointly formed another branch, indicating a closer phylogenetic relationship between *P. patens* and *S. fallax* than to liverworts. Notably, Clade V and Clade VI each contained angiosperm proteins together with pteridophyte AMT2 proteins (SmAMT2;2 and SmAMT2;1). In both trees, members from each plant lineage clustered together within the same clade. We further constructed a phylogenetic tree incorporating the 368 AMT proteins together with two bacterial AmtB proteins. The tree showed that AMT1 and AMT2 formed two distinct clades, with the bacterial AmtB proteins clustering within the AMT2 clade (Figure S1).

Gene duplication analysis revealed that small-scale duplication (SSD), particularly dispersed duplication (DSD), was the primary mechanism driving gene expansion in both *AMT1* and *AMT2* families across the plant kingdom. Notably, whole-genome duplication (WGD) contributed significantly to gene expansion in angiosperms. To assess selection pressure on *AMT* homologous gene pairs, we calculated the numbers of nonsynonymous (Ka) and synonymous (Ks) substitutions and the Ka/Ks ratios (*AMT1* genes in *Porphyra umbilicalis* were all single-copy genes, so no selection pressure analysis was conducted in it). For most gene pairs, the Ka/Ks ratios were below 1. Two exceptions (*GmAMT1;3* vs. *GmAMT1;4*, *PpeAMT2;4* vs. *PpeAMT2;6*) were noted (Figure S2).

### 2.3. Identification, Distribution, and Evolution of AMT Genes in the U’s Triangle

**Figure 3:**
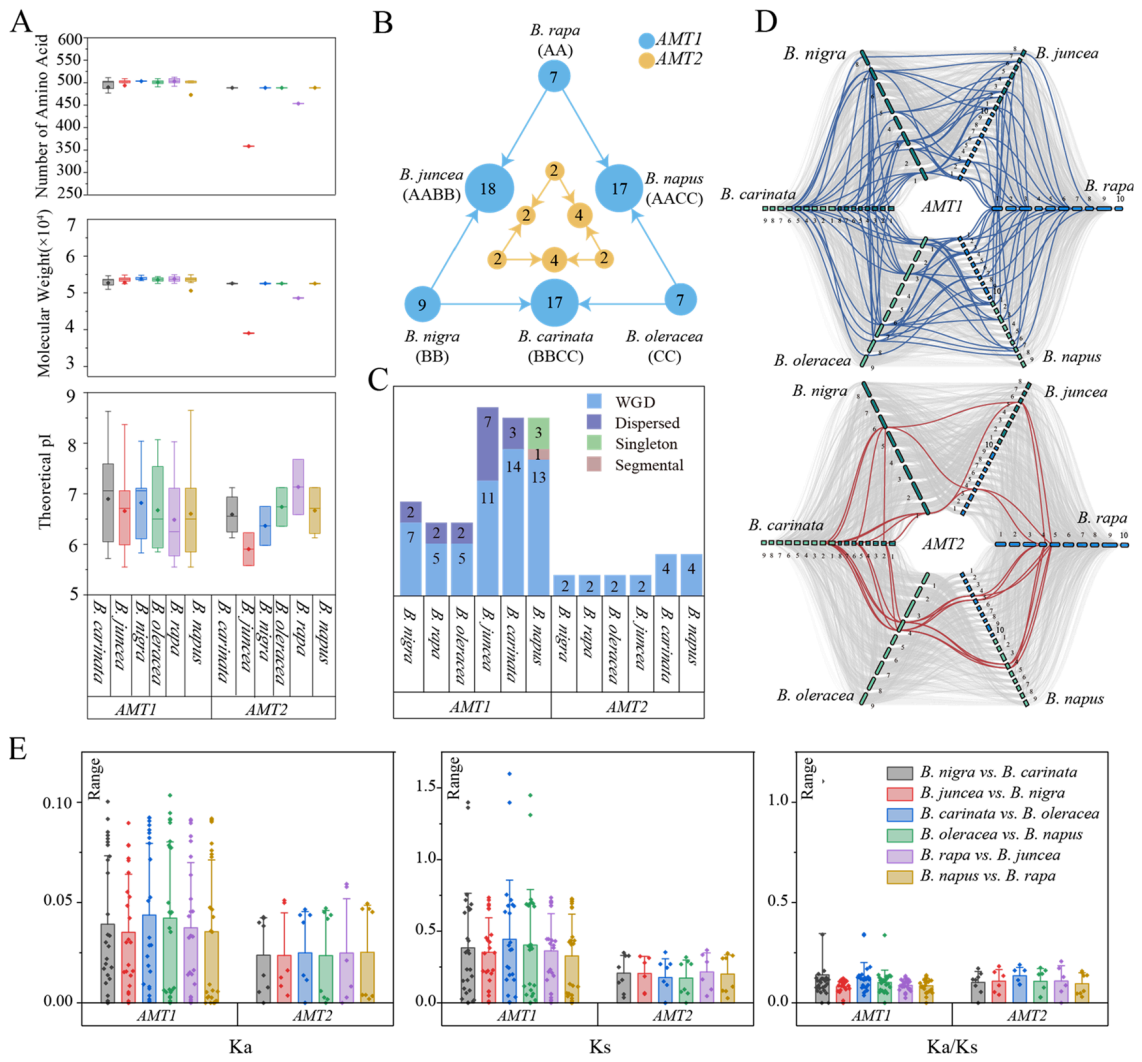
(**A**) Number of amino acids, molecular weight and isoelectric point of *AMT* family in the U’s triangle. (**B**) The number of *AMT* genes in six U’s triangle species. (**C**) Replication types of *AMT* family in the U’s triangle. WGD: genome-wide duplication; tandem: tandem duplication; proximal: proximal duplication; dispersed: dispersed duplication; singleton: single gene. (**D**) Linear relationship diagram of *AMT* family in the U’s triangle. (**E**) Selection pressure analysis of collinear gene pairs of *AMT* genes in U’s triangle.

We identified 91 *AMT* genes (75 *AMT1s* and 16 *AMT2s*) from six *Brassica* species (*B. rapa*, *B. juncea*, *B. nigra*, *B. carinata*, *B. oleracea*, and *B. napus*) and named them according to their chromosomal location (Table S1). Physicochemical property analysis revealed that the 91 AMT proteins ranged from 195 to 575 amino acids in length (average: 487 aa), with molecular weights ranging from 20,851 to 66,832 Da (average: 52,176 Da) and isoelectric points (pI) ranging from 5.55 to 8.65 (average: 6.68) (Figure 3A). Subcellular localization analysis predicted that all 91 AMT proteins were localized to the plasma membrane. In the *AMT1* family, the number of genes in allotetraploids was equal to or greater than the sum of those in their diploid parents (Figure 3B). The *AMT2* family exhibited a similar pattern, with the exception of *B. juncea*, where the number of *AMT2* genes was lower than the sum of its diploid parents.

Collinearity analysis showed that the 75 *AMT1s* and 16 *AMT2s* exhibited collinear relationships among the six U’s triangle species (Figure 3C,D). WGD was the primary driver of expansion for both the *AMT1* and *AMT2* family in the U’s triangle, whereas DSD also contributed to *AMT1* family expansion. For *AMT1*, five orthogroups were detected in *A.thaliana* and subgenomes of the six U’s triangle species, each corresponding to one of the five *AtAMT1* members (*AtAMT1;1*-*AtAMT1;5*). Orthogroup OG0000004 (corresponding to *AtAMT1;2*) was the most conserved group. No WGD-derived redundant copies were retained for this orthogroup. In *B. juncea*, the A subgenome showed evidence of selective sweep, whereas in *B. carinata*, specific gene loss occurred in the B subgenome after its formation. The number of WGD-derived *AMT1* genes in allotetraploids was lower than the total *AMT1* genes in their diploid parents, with losses of five genes in *B. juncea*, two in *B. carinata*, and one in *B. napus* (Table S4). Chromosomal segmental exchanges were detected in association with some *AMT1* genes. All Ka/Ks ratios were below 1 (Figure 3E). Higher Ks values were observed in *AMT1* genes compared with *AMT2* genes.

### 2.4. Regulatory Mechanism in B. napus

**Figure 4:**
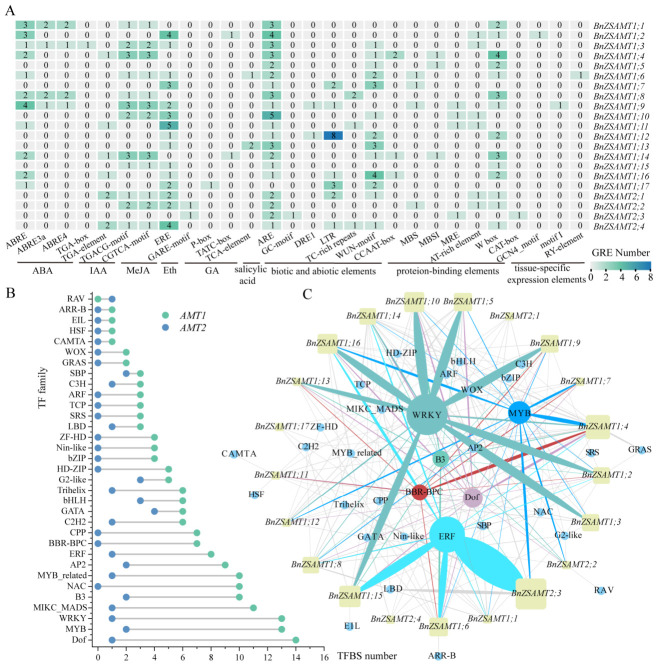
(**A**) Predicted *cis*-acting elements in the 1500 bp upstream regions of *BnAMTs*. The color scale indicates the number of elements per gene, ranging from 0 (white) to 8 (dark blue). (**B**) Number of transcription factor binding sites (TFBS) identified in *BnAMT* promoters across 33 TF families. The X-axis represents the number of TFBS per TF family. (**C**) Regulatory network of *BnZSAMT* genes and their interacting TF. Square nodes represent *BnZSAMT* genes; circular nodes denote TF families. Node size is proportional to connectivity: larger squares indicate *BnZSAMT* genes regulated by multiple TF families; larger circles denote TF families regulating more genes. Line thickness indicates interaction strength.

We predicted *cis*-regulatory elements (CREs) in the 1500 bp upstream regions of 21 candidate *BnZSAMT* genes (Figure 4A, Table S5). A total of 2468 CREs, representing 80 distinct types, were identified. Among the 15 hormone-responsive element types, ABA-responsive (11 genes), MeJA-responsive (12 genes), and ethylene-responsive (15 genes) elements were frequently detected. In addition, six abiotic stress-responsive element types were identified, including anaerobic (ARE; 20 genes) and wound-responsive (WUN-motif, 13 genes) elements. Notably, the *AMT2* family lacked several elements that were present in *AMT1*, including ABA-responsive (ABRE, ABRE3a, ABRE4), salicylic acid-responsive (TCA-element), and cold- and salt-stress-responsive (DRE1) elements. We also identified 1024 potential transcription factor binding sites, belonging to 33 transcription factor families (Figure 4B,C, Table S6). The five families with the highest numbers of binding sites were ERF (19 genes), BBR-BPC (19 genes), B3 (19 genes), MYB (18 genes), and WRKY (15 genes). Specific *BnAMT* genes appeared to be associated with particular transcription factor families. For example, *BnAMT1;15* and *BnAMT2;3* both contained potential ERF family binding sites.

### 2.5. Expression Profile in B. napus

**Figure 5:**
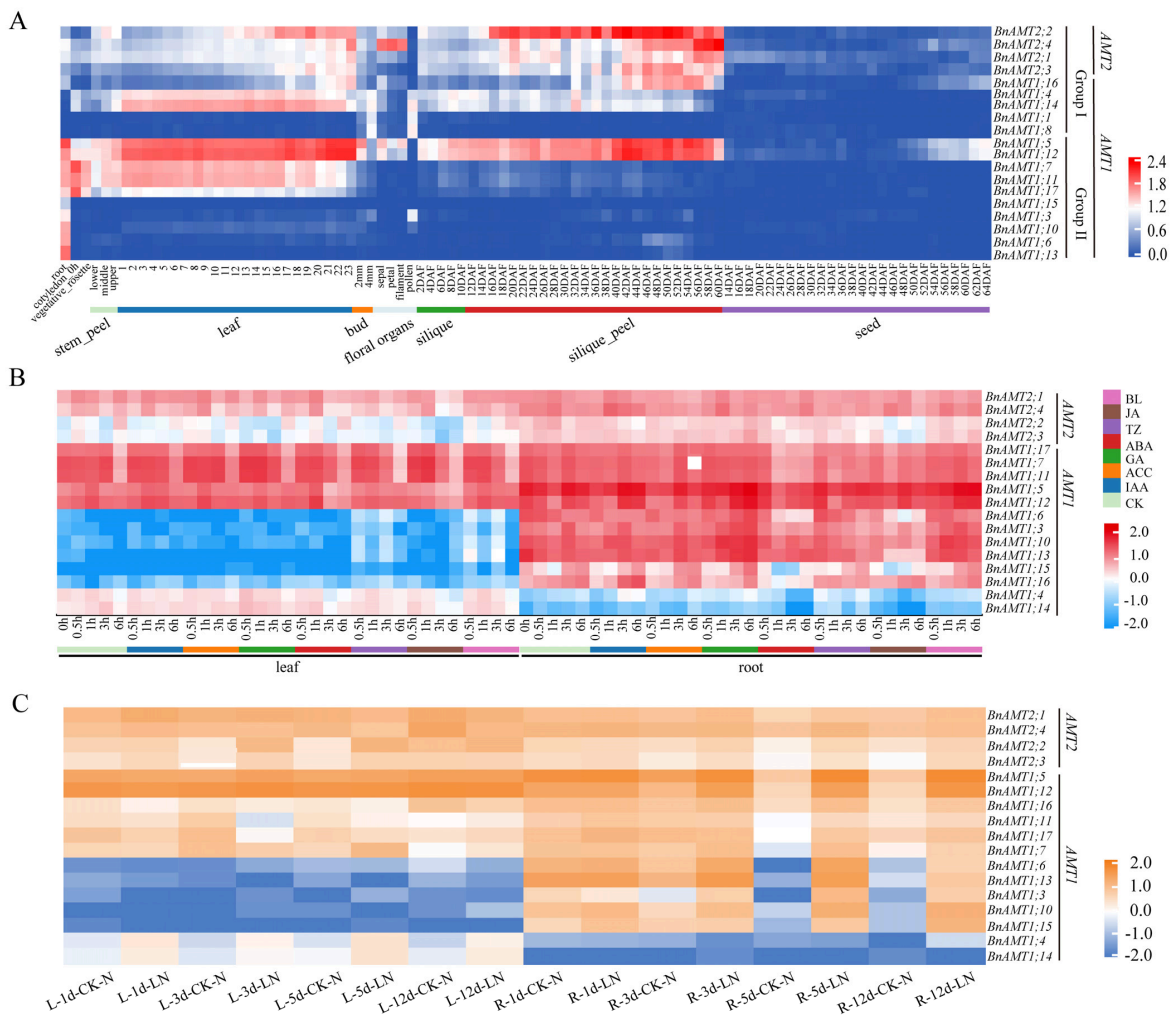
(**A**) Expression pattern of *BnAMTs* across different developmental stages of ZS11. (**B**) Expression profile of *BnAMTs* under hormone inductions. CK: control, IAA: indole acetic acid, ACC: 1-aminocyclopropane carboxylic acid, GA: gibberellin, ABA: abscisic acid, TZ: trans-zeatin, JA: methyl jasmonate, BL: brassinolide, h: hour. (**C**) Expression profile of *BnAMTs* under low nitrogen stress. L = leaf, R = root, d = day, CK = control check. The gradient bar represents log10 expression value: blue represents low expression level, and red or orange represents high expression level. The expression of *BnAMTs* in ZS11 (**A**) was represented by TPM (Transcripts Per Million) value, and the expression of *BnAMTs* under hormone inductions (**B**) and low nitrogen stress (**C**) was represented by FPKM (Fragments Per Kilobase per Million) value.

We characterized the spatiotemporal expression patterns of the 21 *BnaAMT* genes in ZS11 tissues and organs across different developmental stages (Figure 5A). With the exception of *BnAMT1;2* and *BnAMT1;9*, the remaining 19 genes showed detectable expression levels (TPM ≥ 1). In Group I, *BnAMT* genes were not expressed in roots; their expression patterns were divided into two subgroups: one was predominantly expressed in chloroplast-containing organs, such as leaves and silique peels, and the other was predominantly expressed in reproductive organs, including buds and pollen. In Group II, genes were highly expressed in roots, and their expression patterns were also divided into two subgroups: one was expressed in chloroplast-containing organs (e.g., leaves or silique peels), whereas the other was specifically expressed in roots. Both *AMT1* and *AMT2* families included members that were predominantly expressed in leaves and silique peels; only a subset of *AMT1* genes were highly expressed in roots.

We next examined the expression of *BnAMT* genes in roots and leaves of ZS11 under seven phytohormone treatments (IAA, ACC, GA, ABA, TZ, JA, and BL) (Figure 5B). Except for four genes, the remaining 17 genes showed detectable expression levels (FPKM ≥ 1). Most *BnAMTs* were insensitive to IAA, ACC, TZ, and BL, with no significant expression changes in either roots or leaves. In contrast, clear expression shifts were observed in roots following GA, ABA, and JA treatments. Specifically, six genes (35.29%) were up-regulated by GA, whereas eight (47.06%) and nine (52.94%) were down-regulated in response to ABA and JA, respectively. In leaves, seven genes (41.18%) were significantly down-regulated after JA treatment.

The nitrogen-responsive expression profile was analyzed at the seedling stage (Figure 5C). Among the 21 genes, 17 showed detectable expression (FPKM ≥ 1), with *BnAMT1;1*, *BnAMT1;2*, *BnAMT1;8*, and *BnAMT1;9* being undetectable. *BnAMT1;3*, *BnAMT1;6*, *BnAMT1;10*, *BnAMT1;13*, and *BnAMT1;15* showed no significant expression change in leaves but were differentially up-regulated in roots at late time points. Conversely, *BnAMT1;4* and *BnAMT1;14* exhibited low expression levels in roots but were up-regulated in leaves.

Notably, *BnAMT1;5*, *BnAMT1;12*, *BnAMT2;2*, and *BnAMT2;4* showed high expression levels across multiple tissues and clearly responded to low-nitrogen stress. *BnAMT1;5* and *BnAMT1;12* were highly expressed in most tissues except seeds and pollen, and responded to most hormone treatments as well as to low-nitrogen treatment in roots. *BnAMT2;2* and *BnAMT2;4* were highly expressed in silique peels and responded to low-nitrogen treatment in leaves (Figure 5). The expression of these four genes was further examined in two natural *B. napus* populations, where they showed higher expression levels in most accessions compared with other *AMT* genes (Figure S3).

### 2.6. Haplotype Analysis of Candidate BnAMTs

**Figure 6:**
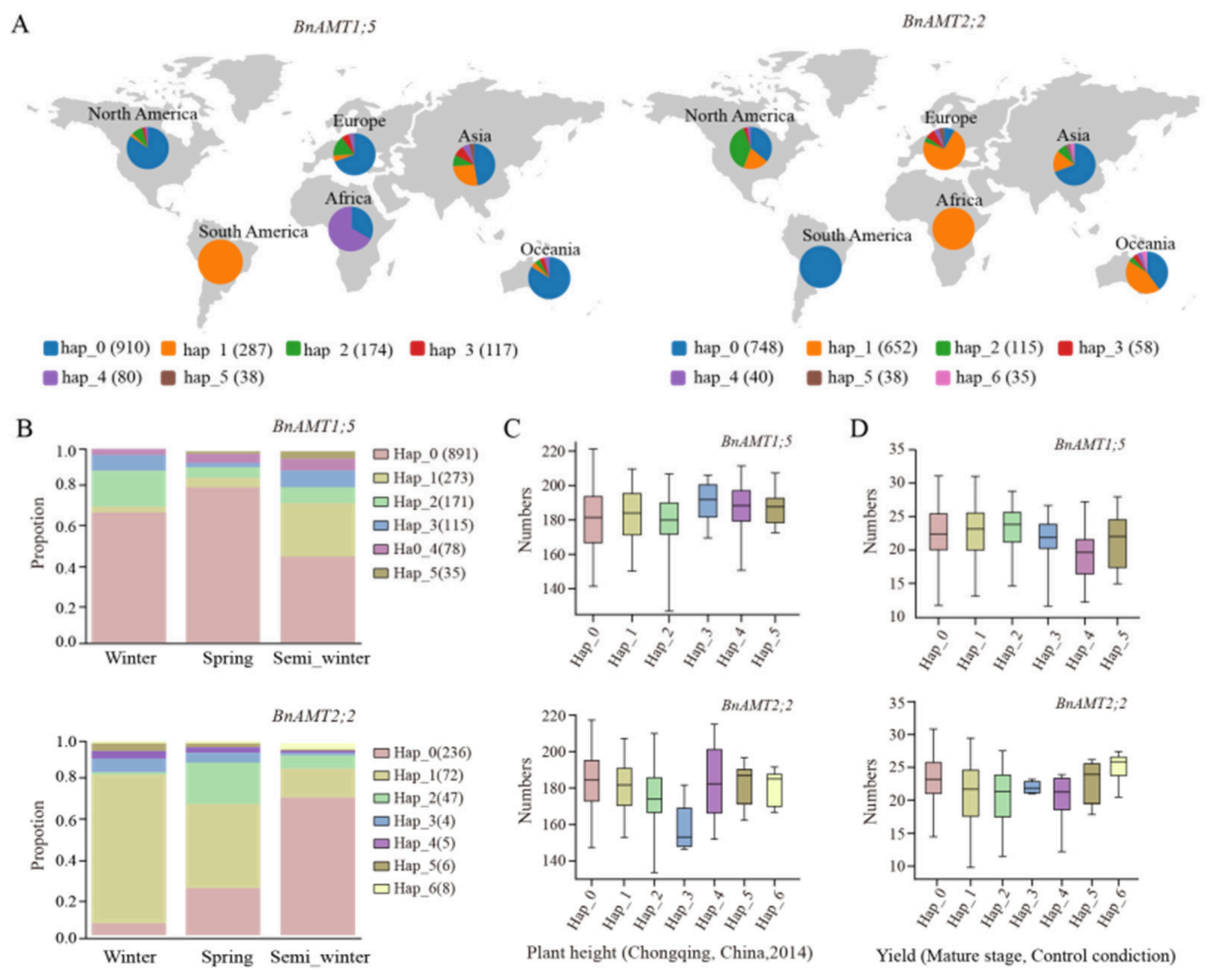
Haplotype analysis of *BnAMT1;5* and *BnAMT2;2*. (**A**) Geographical distribution map of different haplotypes. (**B**) Distribution map of different ecological types. (**C**) Plant height of different haplotypes. (**D**) Yield of different haplotypes.

We performed haplotype analysis of the four selected genes (*BnAMT1;5*, *BnAMT1;12*, *BnAMT2;2*, and *BnAMT2;4*) to examine their associations with plant height and yield. *BnAMT1;12* and *BnAMT2;4* showed no significant correlation with either trait; therefore, subsequent analyses focused on *BnAMT1;5* and *BnAMT2;2* (Figure 6).

*BnAMT1;5* contained 20 mutation sites and formed six haplotypes (Hap_0 to Hap_5). Hap_0 was the dominant haplotype in *B. napus* germplasm from Asia, Europe, Australia, and North America. Functional correlation analysis showed that the Hap_0 population had an average plant height of 180.52 cm, which was higher than that of Hap_2 but lower than those of the other haplotypes. The average yield of the Hap_0 population was lower than that of Hap_1 and Hap_2 but higher than those of the remaining haplotypes.

*BnAMT2;2* contained 40 mutation sites and formed seven haplotypes (Hap_0 to Hap_6). Hap_0 and Hap_1 were the major haplotypes. The Hap_0 population was mainly composed of semi-winter and spring ecotypes distributed in Asia, Australia, North America, and South America. In contrast, the Hap_1 population was predominantly composed of winter and spring ecotypes distributed in Europe, Africa, and Australia. Correlation analysis showed that the Hap_0 population had the highest average height (184.17 cm). Its average yield was lower than that of Hap_6 but higher than those of the other haplotypes.

## 3. Discussion

In this study, 368 *AMT* genes were identified in 35 species and divided into two families (*AMT1*, *AMT2*). We observed an expansion of *AMT1* copy number in algae, which may reflect a pre-adaptive response to the low-nitrogen (L-N) conditions encountered during terrestrial colonization [34]. This finding corroborates previous studies that green algae possess multiple *AMT1s* but lack *AMT2* [35], and we confirm this pattern across a broader range of algal species. The presence of *AMT2* genes in *Klebsormidium* lends further support to the hypothesis that the *AMT2* family originated from bacterial *AmtB* genes via horizontal gene transfer (HGT) [36]. As *Klebsormidium* represents a crucial evolutionary node in the transition from aquatic to terrestrial plants [35], the HGT event was proposed to occur during algal colonization on land. Notably, the *AMT2* gene number peaked in bryophytes, a pattern consistent with the relatively low abundance and activity of membrane transporters previously reported in this lineage [37]. This observation supports the hypothesis that bryophytes expanded their *AMT2* repertoire to compensate for intrinsically low ammonium uptake efficiency.

In this study, most AMTs were predicted to be localized to the plasma membrane, consistent with previous findings in *Arabidopsis* [38] and Chinese cabbage [39], which established the role of AMTs in transmembrane ammonium transport. Moreover, a few AMTs were located in mitochondria, chloroplasts, and the cytoplasm, as also reported in several earlier studies. For example, PutAMT1;1 from the salt-tolerant grass *Puccinellia tenuiflora* was localized not only at the plasma membrane but also at the nuclear periphery and potentially in the endoplasmic reticulum (ER) [40]. Among the AMTs examined, only AtrAMT2;3 was predicted to be localized in the vacuole, where it may sequester excess ammonium to prevent toxicity and remobilize it under nitrogen-limited conditions. These findings suggest that AMTs function not only in ammonium uptake but also in intracellular transport and homeostasis. Conserved motifs previously identified across the AMT/MEP/Rh superfamily (FMAFYAF, FQFAAT, WxWGGG, GGYVH, WNGFNG, and AIHGEE) are largely recapitulated in the eight conserved sequences we detected, underscoring the structural conservation of *AMTs* from bacteria to plants and animals [41]. Several C-terminal motifs identified in this study are consistent with reports that the activity of some plant *AMT1s* is regulated by post-translational phosphorylation of a conserved threonine residue within their C-terminal tails [42].

Regarding the mechanisms underlying *AMT* gene expansion, WGD events are prevalent in the U’s triangle, whereas SSD are more widespread across other plants. This contrast suggests that WGD conferred a selective advantage during allotetraploidization in *Brassica*. This aligns with observations in poplar, soybean, and tomato, where WGD was the major expansion mechanism [43,44]. Gene loss frequently follows WGD in plant genomes, serving to resolve redundancy and eliminate dispensable or deleterious copies [10]. This phenomenon likely explains the loss of two *BjuAMT2* genes in our study.

Beyond copy-number variation and structural conservation, the regulatory networks governing *AMT* expression are equally critical for understanding their impact on nitrogen use efficiency (NUE). Recent studies have established that nutrient signaling crosstalk, particularly involving the *SnRK1* energy-sensing pathway and the nitrogen–iron (N-Fe) balance module, plays a central role in coordinating nitrogen assimilation with plant metabolism [45,46]. Our results show that *BnAMT1;5* and *BnAMT2;2* haplotypes correlate with plant height and yield, and that *BnAMT* expression responds to low-nitrogen and hormonal signals. These findings fit well within this regulatory framework. We propose that AMT-mediated ammonium uptake may be fine-tuned by these broader nutrient signaling networks, integrating nitrogen availability with whole-plant developmental programs. Future investigations into the genetic interplay between *AMT* genes and these regulators in *Brassica napus* may offer actionable targets for breeding nitrogen-efficient varieties.

The four candidate genes identified here represent promising targets for improving nitrogen utilization in crops. In poplar, overexpression of *PdAMT1.1* significantly improves nitrogen use efficiency [47]. Similarly, overexpression of *OsAMT1;1* in rice increases grain weight, yield, and nitrogen uptake efficiency compared to the wild type [48]. In European black poplar, association analysis linked two significant SNP loci (SNP2 and SNP7) to growth under both nitrogen-deficient and nitrogen-sufficient conditions [49]. In alfalfa, the high-NUE cultivar 6010 showed higher root expression of both *AMT* and *NRT2* genes than the low-NUE cultivar Longdong, with expression positively regulated by nitrogen availability [50]. This indicates that *AMT* and *NRT* genes act synergistically to enhance NUE. Collectively, these findings highlight that modulating gene expression, screening key SNP loci, and exploiting polygenic interactions represent promising genetic strategies for improving NUE in agriculture. While our expression analyses have provided valuable clues for candidate gene selection, transcriptomic data alone are insufficient to definitively determine gene function. Further functional validation, such as RT-qPCR and mutant phenotypic analysis, is required to confirm the biological functions of the candidate *BnAMT* genes identified in this study.

In conclusion, our results lay a solid foundation for future studies on the evolution and functional characterization of the *AMT* gene family, and contribute to the long-term goal of improving low-nitrogen tolerance and nitrogen use efficiency in *Brassica napus*.

## 4. Materials and Methods

### 4.1. Identification of AMT Genes

To elucidate the distribution and evolutionary patterns of the AMT gene family in land plants, we selected representative species based on the species tree available in the Phytozome (https://phytozome-next.jgi.doe.gov/, accessed on 1 March 2026) and NCBI databases (https://www.ncbi.nlm.nih.gov/datasets/genome/, accessed on 1 March 2026). The topology of the phylogenetic tree shown in Figure 1A was adopted from the Phytozome database. Specifically, we chose 2–3 representative species from each major lineage, ranging from aquatic plants (Rhodophyta and Chlorophyta) to terrestrial plants, to cover as many major branches of the plant kingdom as possible. Based on this sampling strategy, we selected 35 species that represent distinct evolutionary lineages within the Archaeplastida and for which whole-genome sequences are available. These included five algal species, three bryophytes, two pteridophytes, two gymnosperms, two basal angiosperms, two monocotyledons, and 19 dicotyledons. The genomic data for these 35 species were obtained from the following databases: Phytozome v13, NCBI, TreeGenes (https://treegenesdb.org/FTP/, accessed on 1 March 2026), and BnTIR (https://yanglab.hzau.edu.cn/bntir, accessed on 1 March 2026) (Table S7). To avoid potential omission of *AMT* homologs, we performed an initial BLASTP (v2.17.0) search using the six *Arabidopsis thaliana* AMT protein sequences (AtAMTs) as queries with a low-stringency threshold (cut-off *p*-value < 1 × 10^−1^) [51] (Table S8). The candidate protein sequences were aligned using MAFFT 7.0 (https://mafft.cbrc.jp/alignment/server/, accessed on 1 March 2026); erroneous or severely missing sequences were removed. To validate the conserved domain architecture, all candidate sequences were submitted to InterProScan v5.78 (https://www.ebi.ac.uk/interpro/search/sequence/, accessed on 3 March 2026) against two databases: Pfam (HMM-based) and CDD (RPS-BLAST) (Table S9). Sequences containing the AMT-specific Pfam domain PF00909 (Ammonium Transporter Family) with an E-value < 1 × 10^−5^ were retained. HMM Search (Table S10) and Reverse BLAST (Table S11) were performed for the verification of *AMT* genes in TBtools software (v2.498). Finally, the candidate genes were systematically named based on their chromosomal locations.

### 4.2. Analysis of AMT Gene Physicochemical Properties and Conserved Motifs

Subcellular localization of the candidate proteins was predicted using Plant-mPLoc (http://www.csbio.sjtu.edu.cn/bioinf/plant-multi/, accessed on 13 March 2026), and the results were visualized as a Sankey diagram. The isoelectric points (pI) and molecular weights of the identified AMT proteins were calculated using the Protein Parameter Calc function in TBtools [52], and the data were visualized with Origin 2024. Protein sequences of candidate *AMT* genes were submitted to the MEME website (https://meme-suite.org/meme/doc/meme.html, accessed on 13 March 2026) for conserved motif prediction, with motif lengths ranging from 6 to 50 amino acids and a maximum of 10 motifs per sequence. Conserved motif analysis and visualization were performed using the Gene Structure View (Advanced) tool in TBtools. The three-dimensional structures of *AMT* proteins were predicted using the SWISS-MODEL website (https://swissmodel.expasy.org/, accessed on 13 March 2026).

### 4.3. Phylogenetic Analysis of AMT Genes

Multiple sequence alignments were performed separately for the *AMT1* and *AMT2* family members using MEGA7 [53]. IQ-TREE 2 [54] was employed to identify the best-fit substitution model and to construct maximum likelihood (ML) phylogenetic trees with 1000 bootstrap replicates. The best-fit model for both subfamilies was Pfam + R7. The phylogenetic trees were edited and visualized using the iTOL website (https://itol.embl.de/, accessed on 23 March 2026), and clades were defined based on branch support values. The same procedure was applied to construct the phylogenetic tree of AMT family genes together with bacterial *AmtB* genes, except that the best-fit substitution model was Pfam + R9.

### 4.4. Analysis of Duplicate Gene Origins and Selection Pressure Calculation for AMT Genes

To facilitate downstream analyses, protein sequences and gene annotation data from the 35 species were formatted to ensure consistent gene identifiers. We then used local BLASTP and the MCScanX (v1.0.0) duplicate gene classifier to analyze the duplication modes of *AMT* genes among the 35 species. The identified duplicate gene pairs were then used to calculate selection pressure using KaKs_calculator 3 [55]. The results were visualized with Origin 2024.

### 4.5. Identification and Physicochemical Property Analysis of AMT Genes in the U’s Triangle

*AMT* genes were identified in the genomes of the U’s triangle species (Table S3). Gene identification, subcellular localization prediction, and physicochemical property calculation were performed using the same methods described in Section 4.1.

### 4.6. Evolutionary Analysis of the AMT Genes in U’s Triangle

Collinearity analysis of *AMT* genes among the six U’s triangle species was performed using MCScanX with default parameters (E-value ≤ 1 × 10^−3^) [56]. Detailed synteny information for each AMT-containing block is provided in Supplementary Table S12. Syntenic relationships were visualized using the JCVI (v1.6.5) Python library with the cscore parameter set to 0.5 [57]. Duplication modes of all *AMT* genes across the six species were determined based on the synteny strengths derived from MCScanX. Orthogroups were identified using OrthoFinder, and selection pressures on the resulting duplicate gene pairs were calculated with KaKs_calculator 3.0. The results were visualized subsequently using Origin 2024.

### 4.7. Characterization of BnAMTs Gene Expression

Potential *cis*-acting elements and transcription factors in the 1500 bp upstream promoter regions of *BnAMT* genes were predicted using the PlantCARE (http://bioinformatics.psb.ugent.be/webtools/plantcare/html/, accessed on 23 March 2026) and PlantTFDB (https://planttfdb.gao-lab.org/, accessed on 23 March 2026) databases. The transcriptional regulatory network was constructed and visualized using Cytoscape 3.9.1 [58]. Transcriptome data for ZS11 were acquired from the BnIR database (https://yanglab.hzau.edu.cn/, accessed on 24 March 2026) to analyze the spatiotemporal expression patterns and responses to seven phytohormone treatments. Transcriptome data under low-nitrogen stress were derived from the root and leaf RNA-seq datasets of ZS11 seedlings [59]. Population-level expression data were obtained from the BnIR database (https://yanglab.hzau.edu.cn/, accessed on 26 March 2026) and the BnaOmics database (https://bnaomics.ocri-genomics.net/, accessed on 26 March 2026) to examine *BnAMT* expression profiles across different *B. napus* accessions.

### 4.8. Haplotype Analysis

Haplotype data for *BnAMT1;5* and *BnAMT2;2* were obtained from the BnVIR database (https://yanglab.hzau.edu.cn/BnVIR, accessed on 28 March 2026). Corresponding phenotypic data for plant height and yield across different haplotypes were retrieved from the same database.

## 5. Conclusions

This study systematically identified 368 *AMT* genes across 35 representative green plant species, elucidating their evolutionary trajectory from aquatic algae to angiosperms. Small-scale duplication (SSD), especially dispersed duplication, drove *AMT* expansion throughout plant evolution, whereas whole-genome duplication (WGD) predominated in U’s triangle species. Purifying selection and conserved motifs maintained functional stability, while lineage-specific variations reflected adaptive strategies to diverse nitrogen environments. Four *BnAMT* genes (*BnAMT1;5*, *BnAMT1;12*, *BnAMT2;2*, and *BnAMT2;4*) were identified as candidates for enhancing nitrogen use efficiency, with haplotype analysis linking *BnAMT1;5* and *BnAMT2;2* polymorphisms to plant height and yield variation in *B. napus*. These findings provide a foundation for understanding *AMT* evolution and offer genetic resources for breeding nitrogen-efficient rapeseed cultivars.

## Data Availability

The original contributions presented in this study are included in the article/Supplementary Materials. Further inquiries can be directed to the corresponding authors.

## Supplementary Materials

The following supporting information can be downloaded at: https://www.mdpi.com/article/10.3390/plants15152334/s1, Figure S1: Phylogenetic tree of *AMT* gene family and bacterial *AmtB* genes; Figure S2: Ka/Ks values of *AMT* repeat gene pairs in 34 species; Figure S3: Expression profile of *BnAMTs* in different populations of *B. napus*; Table S1. *AMT* gene family members in representative species of plant kingdom; Table S2: The distribution of *AMT* genes in different clades of phylogenetic tree; Table S3: Reference genome information of *Brassica* species in U’s triangle; Table S4: The orthogroups of *AMT1* genes in *A. thaliana* and *Brassica* species of U’s triangle; Table S5: Number of *cis*-acting elements in the promoter regions of *AMT* gene family in *Brassica napus*; Table S6: Number of transcription factor binding sites in the promoter regions of *AMT* gene family in *Brassica napus*; Table S7: Reference genome information of 35 species identified in the plant kingdom; Table S8: BLASTP results against *Arabidopsis thaliana* AMTs in 35 species of plant kingdom; Table S9: List of 368 *AMT* sequences validated by Pfam and CDD domains; Table S10: HMM search of PF00909 domain in 35 representative Archaeplastida speciese; Table S11: Reverse blast of 368 *AMT* genes in *A. thaliana*; Table S12. MCScanX-based syntenic blocks of *AMT* genes in U’s triangle species.
